# Similarities in trabecular hypertrophy with site-specific differences in cortical morphology between men and women with type 2 diabetes mellitus

**DOI:** 10.1371/journal.pone.0174664

**Published:** 2017-04-06

**Authors:** Janina M. Patsch, Sazan Rasul, Florian A. Huber, Karoline Leitner, Anita Thomas, Roland Kocijan, Stephanie Boutroy, Michael Weber, Heinrich Resch, Franz Kainberger, Claudia Schüller-Weidekamm, Alexandra Kautzky-Willer

**Affiliations:** 1 Department of Biomedical Imaging and Image-Guided Therapy, Division of General Radiology and Pediatric Radiology, Medical University of Vienna, Vienna, Austria; 2 Department of Biomedical Imaging and Image-Guided Therapy, Division of Nuclear Medicine, Medical University of Vienna, Vienna, Austria; 3 Department of Internal Medicine III, Division of Endocrinology and Metabolism, Gender Medicine Unit, Medical University of Vienna, Vienna, Austria; 4 Department of Internal Medicine II, Karl Landsteiner Institute for Rheumatology and Gastroenterology, St. Vincent Hospital Vienna, Vienna, Austria; 5 INSERM Research Unit 831, Université de Lyon, Lyon, France; 6 Department of Biomedical Imaging and Image-Guided Therapy, Medical University of Vienna, Vienna, Austria; 7 Department of Biomedical Imaging and Image-Guided Therapy, Division of Neuroradiology and Musculoskeletal Radiology, Medical University of Vienna, Vienna, Austria; Oklahoma State University, UNITED STATES

## Abstract

The goal of our study was to investigate interactions between sex and type 2 diabetes mellitus (T2DM) with regard to morphology of the peripheral skeleton. We recruited 85 subjects (mean age, 57±11.4 years): women with and without T2DM (n = 17; n = 16); and men with and without T2DM (n = 26; n = 26). All patients underwent high-resolution, peripheral, quantitative, computed tomography (HR-pQCT) imaging of the ultradistal radius (UR) and tibia (UT). Local bone geometry, bone mineral density (BMD), and bone microarchitecture were obtained by quantitative analysis of HR-pQCT images. To reduce the amount of data and avoid multi-collinearity, we performed a factor-analysis of HR-pQCT parameters. Based on factor weight, trabecular BMD, trabecular number, cortical thickness, cortical BMD, and total area were chosen for post-hoc analyses. At the radius and tibia, diabetic men and women exhibited trabecular hypertrophy, with a significant positive main effect of T2DM on trabecular number. At the radius, cortical thickness was higher in diabetic subjects (+20.1%, p = 0.003). Interestingly, there was a statistical trend that suggested attenuation of tibial cortical hypertrophy in diabetic men (cortical thickness, p_interaction_ = 0.052). Moreover, we found an expected sexual dichotomy, with higher trabecular BMD, Tb.N, cortical BMD, Ct.Th, and total area in men than in women (p≤ 0.003) at both measurement sites. Our results suggest that skeletal hypertrophy associated with T2DM is present in men and women, but appears attenuated at the tibial cortex in men.

## Introduction

Fragility fractures are increasingly recognized as a skeletal secondary complication of type 2 diabetes mellitus (T2DM) [1–4]. Although subjects with T2DM carry a high risk of falls due to impaired eyesight, polyneuropathy, and fatty atrophy of the musculature, these factors have been shown to be insufficient to explain the disproportionately high rate of fractures [5]. Currently, the pathogenesis of diabetic bone disease and associated fragility fractures is not sufficiently understood. Bone mineral density (BMD)—as measured by dual-energy, x-ray absorptiometry (DXA) or quantitative computed tomography (QCT)—is typically high to normal or only mildly reduced in patients with T2DM [6]. Potential explanations for the paradoxical positive association of high BMD and fragility fractures include microarchitectural and matrix-based causes, such as cortical porosity [7, 8], and deposition of advanced glycation end products (AGEs) [9]. On a cellular level, diabetic bone disease is characterized by low bone turnover [10, 11], and there are also numerous suggestions of a significant imbalance of the WNT/SOST/PTH pathway, possibly through osteocyte dysfunction [12, 13].

High-resolution, peripheral, quantitative computed tomography (HR-pQCT) has been used by several researchers to study bone geometry, compartment-specific volumetric bone mineral density (vBMD), and bone microarchitecture—including cortical porosity—of the ultradistal extremities in subjects with T2DM [7, 8, 11, 14, 15].

In the past decade, HR-pQCT has been validated with bone biopsies (i.e., the gold standard method for the quantitative assessment of bone microarchitecture), DXA, and QCT of the axial and peripheral skeleton [16–19]. HR-pQCT has provided key insights into the morphology and pathophysiology of diabetic bone disease in elderly subjects. Poor cortical bone quality, particularly high cortical porosity, has been reported by several researchers ([7, 8, 14]. At the same time, trabecular BMD and trabecular microarchitecture, as determined by HR-pQCT, appear to be stable or even relatively high in subjects with T2DM [7, 15]. The above-mentioned microstructural findings have been well documented in postmenopausal diabetic women, but only few studies have investigated bone microarchitecture in men with T2DM [15]. Recently, Paccou et al. reported unfavorable associations between bone quality and T2DM in men. Specifically, they found cortical bone quality to be pathologically altered in both elderly men and women, with more pronounced findings in men.

In the general, non-diabetic population, sex-specific differences in bone geometry, bone mineral density (BMD), and bone microarchitecture are well recognized and viewed as the causes for the clinical differences in fracture prevalence between men and women [20, 21]. Using HR-pQCT, it has been confirmed that young men have larger bones with higher trabecular bone volume and higher trabecular thickness than young women [22]. With aging, trabecular bone volume decreases proportionately in both men and women, but trabecular microarchitecture remains better preserved in men. Cortical thickness appears to be comparable in younger and middle-aged men and women, but over time, especially at older ages, thickness decreases are larger in women [22]. Interestingly, the cross-sectional area of long bones increases with normal aging by periosteal apposition in both sexes [20, 23, 24]. Of importance, metabolic bone diseases can alter this physiologic pattern (e.g., as seen in male idiopathic osteoporosis) [25]. Considering the importance of age- and sex-specific skeletal differences for the modulation of fracture risk in the general population, and accumulating evidence for impaired bone quality in women and men with T2DM, we designed a study to investigate the interactions between sex and T2DM in the peripheral adult skeleton.

## Patients and methods

### Subjects

Thirty-three women and 52 men were recruited into one of four groups: women with type 2 diabetes mellitus (WT2DM; n = 17); women without type 2 diabetes mellitus (WCo; n = 16); men with type 2 diabetes mellitus (MT2DM; n = 26); and men without type 2 diabetes mellitus (MCo; n = 26). Diabetic subjects were recruited from the Endocrine Outpatient Unit of the Department of Internal Medicine III of the Medical University of Vienna, Austria. Healthy women (WCo) were recruited by the VINFORCE study group/Department of Internal Medicine II, St. Vincent Hospital Vienna, Austria. Healthy (i.e., non-diabetic) men (MCo) were recruited as part of the STRAMBO study, an epidemiologic cohort study conducted by the Université de Lyon, France [26]. The study was approved by the ethics committees of the Medical University of Vienna, the St. Vincent Hospital Vienna, and the Université de Lyon. All participants gave written, informed consent.

Inclusion criteria for all subjects were age 40–75 years and written, informed consent. Diabetic men and women had to be treated with standard antidiabetics and have HbA_1_C values ranging from 6–10%. Women had to be postmenopausal.

Current or previous use of rosiglitazone, steroids, antiepileptic drugs, vitamin K antagonists, bisphosphonates, fluorides, PTH, strontium ranelate, raloxifen, denosumab, and calcitonin were defined as exclusion criteria. Severe hepatic and/or renal failure, active malignancy, or a history of malignancy, and pregnancy excluded subjects from study participation.

### HR-pQCT imaging

All subjects underwent HR-pQCT imaging of the non-dominant ultradistal radius and the left tibia (XtremeCT; Scanco Medical AG, Brüttisellen, Switzerland). Diabetic subjects and female non-diabetic controls were scanned at the Medical University of Vienna, Austria. Male non-diabetic controls underwent imaging in Lyon, France. To highlight cross-calibration validity and exclude a multi-center bias, an additional seven non-diabetic men were scanned and analyzed in Vienna. The two HR-pQCT scanners (first-generation devices) used in this study were cross-calibrated, as published by Burghardt et al. [27]. The identical standard *in vivo* protocol [22, 28] was used in both Vienna and Lyon, and was defined by the following settings: 60kVP; 900 μA; and 100 ms integration time. In case of local fracture history, the contralateral extremity was scanned. After the acquisition of a local scout view, a reference line was placed on the joint surface of the radius and tibia. From the reference line, fixed offsets were used to define the scan region (radius offset: 9.5mm; tibia offset: 22.5mm). The final scan volume covered a length of 9.02mm, corresponding to 110 slices. The nominal resolution of HR-pQCT images was isotropic (82 x 82 x 82 μm). The effective dose was < 4 μSv, the scan time was < 3 minutes per scan.

### Image analysis

For quality control, visual semiquantitative motion grading was performed prior to quantitative image analysis. According to the criteria established by Pialat et al., only scans reaching image-quality grades 1–3 were used for quantitative analyses [29]. HR-pQCT images were segmented semi-automatically and analyzed with the standard protocol provided by the manufacturer of the device. Semiautomatic contours were reviewed for accuracy, and manual adjustment was limited to clear contour deviations from the anatomical periosteal boundaries. Volumetric BMD and morphometric parameters were obtained for trabecular and cortical bone [30]. Trabecular bone volume fraction (BV/TV) was derived from trabecular BMD using an assumed density of 100% for compact mineralized bone (1200 mg HA/cm^3^) and background marrow (0 mg HA/cm^3^). Trabecular number and the standard deviation of inter-trabecular distances were calculated by distance transformation [31]. Trabecular thickness and trabecular separation were derived from trabecular BMD and trabecular number [32]. Cortical thickness was obtained by annular approximation [30, 33].

In addition, HR-pQCT images were reviewed for the presence of vascular calcifications, which were defined as linear or tubular hyperdensity zones of circular, semi-circular, or crescent-like shape, which corresponded to the anatomical territory of the anterior tibial artery, the posterior tibial artery, the radial artery, the ulnar artery, the interosseous branches, or smaller intramuscular or subcutaneous arterioles [34]. Skin calcifications or other non-vascular soft tissue calcifications were not included.

### Statistical analysis

All statistical analyses were performed using IBM SPSS Statistics version 22. Metric data were described using means +/- standard deviation (SD) if normally distributed or, in case of highly skewed data, medians [min; max]. Categorical data were presented using absolute numbers and percentages. As there was a large number of highly correlated measures obtained for the tibia and radius, principal axis factor analyses (FA) was used to reduce the number of necessary statistical tests and to minimize an error of the first type. Only parameters with the highest loading within a factor were used for subsequent analyses.

In order to test the moderation effect of sex on the effect of diabetes, two-way analyses of variance were used.

Unpaired student t-tests were used to determine differences in age and laboratory data. To compare the percentage of male and female patients with and without calcifications, a Fisher’s exact test was applied. Due to the limited sample size, we refrained from using multiplicity corrections to avoid decreasing power. In order to rule out a multi-center bias, a small subset of non-diabetic male participants from Lyon was compared with non-diabetic men from Vienna who were not part of the original study. A p-value equal to or below 5% was considered to indicate significant results.

## Results

### Subject characteristics

Demographics and clinical characteristics are given in Table 1. There were no significant differences in age. Laboratory data were available only from subjects with T2DM and not from non-diabetic controls. Comparing diabetic men and women, there were no significant differences in fasting blood glucose (p = 0.804), HbA1c-levels (p = 0.411), serum insulin (p = 0.730), PTH (p = 0.126), and 25-OH-vitamin D (p = 0.074). Serum creatinine was higher in diabetic men, but remained within normal limits (1.0 mg/dl; p = 0.013). As determined from visual assessment of HR-pQCT scans by a board-certified radiologist (JMP), there were no significant differences between lower leg vascular calcification frequencies in diabetic men (50% with calcifications) and women (42.4% with calcifications, p = 0.607). Likewise, at the upper extremity, there were no significant differences between vascular calcification frequencies in diabetic men (19.2% with calcifications) and women (9.1% with calcifications, p = 0.284).

**Table 1:** Demographics and clinical characteristics.

	WT2DM (n = 17)	WCo (n = 16)	MT2DM (n = 26)	MCo (n = 26)
**Age (years)**	58 ± 14.29	55 ± 8.10	57 ± 11.11	57 ± 11.19
**Height (m)**	1.62 ± 0.07	1.69 ± 0.06	1.73 ± 0.06	1.71 ± 0.09
**BMI (kg/m**^**2**^**)**	33.47 ± 4.95	24.19 ± 3.50	29.65 ± 4.35	26.01 ± 2.92
**HbA1c (%)**	7.91 ± 1.52	n/a	7.54 ± 1.15	n/a
**Blood glucose**	148.21 ± 45.05	90 ± 6.93 (n = 3)	144.68 ± 38.80	n/a
**Insulin**	9.85 ± 5.25	n/a	10.82 ± 8.83	n/a
**PTH**	50.90 ± 27.05	n/a	40.05 ± 15.09	n/a
**25-OH-Vit D**	33.34 ± 15.96	n/a	47.38 ± 23.79	n/a
**Calcitriol**	29.31 ± 12.34	n/a	41.79 ± 13.06	n/a
**Folic acid**	18.91 ± 8.47	n/a	20.50 ± 7.11	n/a
**Vitamin B12**	282.31 ± 193.92	n/a	299.35 ± 182.87	n/a
**Calcium**	2.40 ± 0.10	n/a	2.46 ± 0.05	n/a
**Creatinine**	0.83 ± 0.15	n/a	0.99 ± 0.21	n/a
**Uric acid**	5.53 ± 0.94	n/a	5.60 ± 1.28	n/a
**Medical history n (%)**				
**Never used tobacco**	8 (53.3%)	n/a	8 (33.3%)	n/a
**Hypertension**	14 (93.3%)	n/a	16 (66.6%)	n/a
**Duration of T2DM (in years)**	8.21 ± 5.82	n/a	7.71 ± 7.12	n/a
**Current medication n (%)**				
**Metformin**	11 (73.3%)	n/a	19 (79.17%)	n/a
**Sulfonylureas**	10 (66.6%)	n/a	9 (37.5%)	n/a
**Insulin-sensitizer**	0	n/a	0	n/a
**Insulin**	1 (6.6%)	n/a	1 (4.17%)	n/a
**DPP-4**	4 (26.6%)	n/a	6 (25%)	n/a
**Pioglitazone**	7 (46.6%)	n/a	14 (58.3%)	n/a

### HR-pQCT

**Fig 1:**
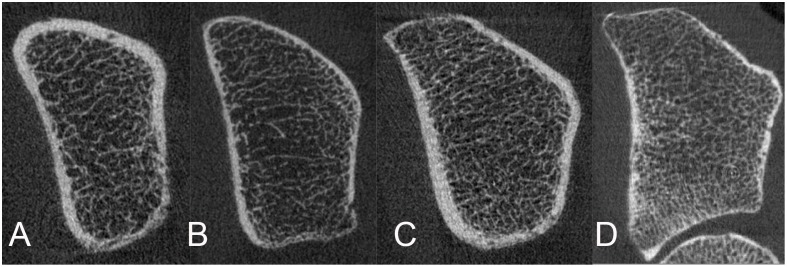
HR-pQCT of the ultradistal radius: Representative images. A) Woman with T2DM. B) Woman without T2DM. C) Man with T2DM. D) Man without T2DM.

**Fig 2:**
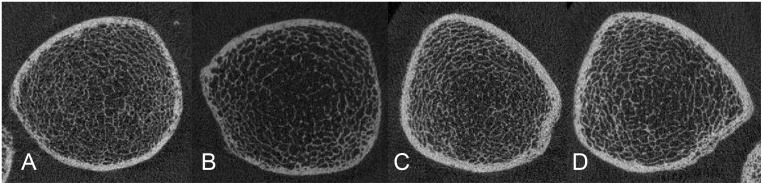
HR-pQCT of the ultradistal tibia: Representative images. A) Woman with T2DM. B) Woman without T2DM. C) Man with T2DM. D) Man without T2DM.

For all four subject groups (i.e., WT2DM, WCo; MT2DM, MCo), means and standard deviations of HR-pQCT parameters are given in Table 2. Moreover, Table 2 provides relative differences in HR-pQCT parameters between subjects with and without T2DM (with separate analyses for men and women). Fig 1 (radius) and Fig 2 (tibia) illustrate bone morphology in men and women with and without T2DM.

**Table 2:** HR-pQCT parameters of the ultradistal radius and the ultradistal tibia in men and women with and without type 2 diabetes mellitus.

	WT2DM (n = 17)	WCo (n = 16)	Mean Relative Difference (%)	MT2DM (n = 26)	MCo (n = 26)	Mean Relative Difference (%)
**Ultradistal Radius**						
**Total area (mm**^**2**^**)**	266±73	247±48	+7.4%	346±50	380±76	-9%
**Cortical area (mm**^**2**^**)**	53.1±12.4	41.2±8.3	+29%	76.3±20.5	67.2±14.9	+13.6%
**Trabecular area (mm**^**2**^**)**	205±70	200±49	+2.6%	263±56	304±73	-13.3%
**Total BMD (mgHA/mm**^**3**^**)**	331±49	291±75	+14%	362±94	326±63	+10.9%
**Cortical BMD (mgHA/mm**^**3**^**)**	865±99	847±77	+2.2%	887±56	848±54	+4.6%
**Trabecular BMD (mgHA/mm**^**3**^**)**	158±30	143±36	+10.3%	193±53	187±36	+3.2%
**Cortical thickness (mm)**	0.79±0.21	0.64±0.17	+24%	0.96±0.30	0.80±0.19	+19.9%
**Bone volume fraction (BV/TV, %)**	0.13±0.02	0.12±0.03	+10.3%	0.16±0.04	0.16±0.03	+3.2%
**Trabecular number (1/mm)**	2.02±0.29	1.75±0.18	+15.6%	2.18±0.23	1.91±0.22	+14.4%
**Trabecular thickness (mm)**	0.07±0.01	0.07±0.01	-4.9%	0.07±0.02	0.08±0.01	-9.8%
**Trabecular separation (mm)**	0.44±0.08	0.51±0.08	-13.4%	0.39±0.05	0.45±0.07	-13.5%
**Trabecular heterogeneity (mm)**	0.19±0.05	0.22±0.05	-16.7%	0.17±0.015	0.19±0.04	-11.1%
**Ultradistal Tibia**						
**Total area (mm**^**2**^**)**	674±90	673±120	+0.2%	861±120	825±135	+4.4%
**Cortical area (mm**^**2**^**)**	109±23	83±15	+30.1%	148±35	147±32	+0.2%
**Trabecular area (mm**^**2**^**)**	555±103	593±106	-6.4%	714±126	672±131	+6.2%
**Total BMD (mgHA/mm**^**3**^**)**	290±42	246±51	+18%	312±64	316±55	-1.4%
**Cortical BMD (mgHA/mm**^**3**^**)**	835±72	807±62	+3.5%	872±55	881±55	-1.0%
**Trabecular BMD (mgHA/mm**^**3**^**)**	168±29	155±27	+8.9%	189±42	183±37	+2.7%
**Cortical thickness (mm)**	1.08±0.26	0.86±0.20	+25.9%	1.28±0.34	1.31±0.27	-2.1%
**Bone volume fraction (BV/TV, %)**	0.14±0.02	0.13±0.02	+8.5%	0.16±0.03	0.15±0.03	+2.8%
**Trabecular number (1/mm)**	2.03±0.35	1.63±0.23	+24.6%	2.26±0.29	1.81±0.26	+25%
**Trabecular thickness (mm)**	0.07±0.01	0.08±0.01	-12.1%	0.07±0.01	0.08±0.01	-18%
**Trabecular separation (mm)**	0.44±0.08	0.54±0.09	-19.4%	0.38±0.06	0.48±0.09	-20.8%
**Trabecular heterogeneity (mm)**	0.19±0.05	0.25±0.06	-22.3%	0.16±0.04	0.22±0.07	-28.3%

Data reduction by factor-analysis yielded four factor groups (‘first level factors’) identical for the radius and tibia parameters. Detailed results for factor-analysis are given in Table 3. Based on the highest factor weight within factor groups (Table 3), four representative HR-pQCT parameters were chosen from each group for selective post-hoc testing. Trabecular BMD (chosen from the factor-group that contained trabecular density, trabecular bone volume fraction, and trabecular thickness), trabecular number (chosen from the factor-group that contained trabecular number, trabecular separation, and trabecular heterogeneity), cortical thickness (chosen from the factor-group that contained cortical area, total density, cortical density, and cortical thickness), and total area (chosen from the factor-group that contained total area, trabecular area, and cortical perimeter). Due to independent information provided by cortical BMD, additional post-hoc testing was performed for cortical BMD as a fifth parameter. The post-hoc choice of HR-pQCT parameters was identical for the radius and tibia (Table 3).

**Table 3:** Factor-analysis of HR-pQCT parameters of the ultradistal radius and the ultradistal tibia. Numbers in columns are factor loadings per parameter (in rows).

	FACTOR			
	1	2	3	4
	**RADIUS**			
**Total area (mm**^**2**^**)**				**.*983***
**Cortical area (mm**^**2**^**)**			**.*878***	
**Trabecular area (mm**^**2**^**)**				**.*966***
**Total BMD (mgHA/mm**^**3**^**)**	**.*525***		**.*730***	
**Cortical BMD (mgHA/mm**^**3**^**)**			**.*793***	
**Trabecular BMD (mgHA/mm**^**3**^**)**	**.*859***	**.*407***		
**Cortical thickness (mm)**			**.*939***	
**Bone volume fraction (BV/TV, %)**	**.*856***	**.*413***		
**Trabecular number (1/mm)**		**.*957***		
**Trabecular thickness (mm)**	**.*947***			
**Trabecular separation (mm)**		**-.*914***		
**Trabecular heterogeneity (mm)**		**-.*862***		
	**TIBIA**			
**Total area (mm**^**2**^**)**				**.*983***
**Cortical area (mm**^**2**^**)**			**.*852***	
**Trabecular area (mm**^**2**^**)**				**.*969***
**Total BMD (mgHA/mm**^**3**^**)**	**.*584***		**.*710***	
**Cortical BMD (mgHA/mm**^**3**^**)**			**.*899***	
**Trabecular BMD (mgHA/mm**^**3**^**)**	**.*886***			
**Cortical thickness (mm)**			**.*910***	
**Bone volume fraction (BV/TV, %)**	**.*885***			
**Trabecular number (1/mm)**		**.*930***		
**Trabecular thickness (mm)**	**.*826***	**-.*457***		
**Trabecular separation (mm)**		**-.*909***		
**Trabecular heterogeneity (mm)**		**-.*929***		

**Fig 3:**
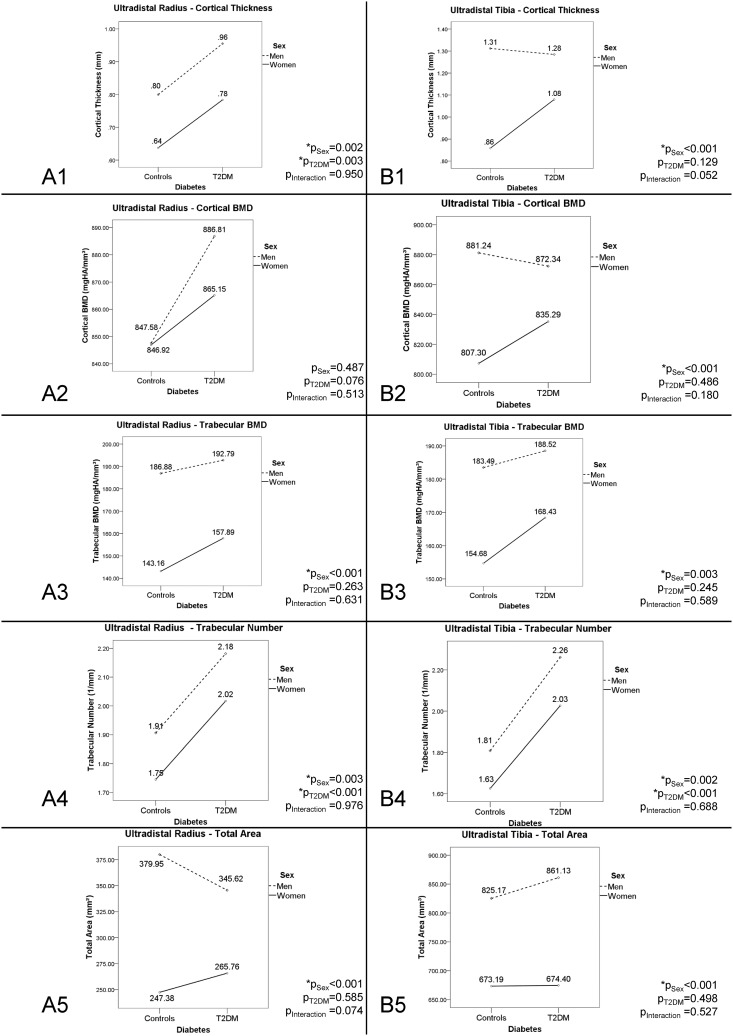
HR-pQCT of the ultradistal radius and tibia in men and women with and without type 2 diabetes mellitus. Means for cortical thickness (in mm), cortical BMD (in mgHA/mm^3^), trabecular BMD (in mgHA/mm^3^), trabecular number (in 1/mm), and total area (in mm^2^). Dashed line represents mean differences between diabetic and non-diabetic men, and continuous line represents mean differences between diabetic and non-diabetic women. p-values are given for differences between men and women (p_sex_), differences between subjects with and without T2DM (pT2DM), and interactions between sex and T2DM (p_interaction_).

At the ultradistal radius, trabecular BMD (+25.8%, p<0.001), trabecular number (+7.4%, p = 0.003), cortical thickness (+21.9%, p = 0.002), and total area (+36.2%, p<0.001) were significantly higher in men than in women. There were no significant sex-specific differences in cortical BMD. Trabecular number (+14.7%, p<0.001) and cortical thickness (+20.1%, p = 0.003) were significantly higher in subjects with T2DM than in non-diabetic subjects. We found a trend toward higher cortical BMD in subjects with T2DM (+3.6%, p = 0.076). Regarding total area, there was a trend toward a significant interaction between sex and T2DM (p = 0.074). For visualization of data (including interactions) and a complete list of p-values, please see Fig 3.

At the ultradistal tibia, trabecular BMD (+13.1%, p = 0.003), trabecular number (+10.4%, p = 0.002), cortical thickness (+34.1%, p<0.001), cortical BMD (+6.8%; p<0.001), and total area (+25.0%, p<0.001) were significantly higher in men than in women. T2DM had a significant association with trabecular number (+24.1%, p<0.001), but no significant associations with trabecular BMD, cortical thickness, cortical BMD, or total area. We found a trend toward an interaction between sex and T2DM for cortical thickness (p = 0.052). There were no significant interactions between sex and T2DM with regard to trabecular BMD, trabecular number, cortical BMD, or total area at the tibia. For visualization of data (including interactions) and a complete list of p-values, please see Fig 3.

There were no significant differences in HR-pQCT parameters between an age-matched subset of healthy male participants scanned in Lyon, France, and healthy men from Vienna, Austria (Table 4), reflecting the validity of our multi-center approach.

**Table 4:** Comparison of male participants without T2DM from two European HR-pQCT sites.

	Healthy male participants (Austria)	Healthy male participants (France)	p-value
**Demographics**			
Age (years)	53.54 ± 7.12	54.04 ± 7.61	0.90
Height (m)	1.73 ± 6.43	1.74 ± 10.33	0.98
Weight (kg)	85 ± 22.34	76.86 ± 9.70	0.42
BMI (kg/m^2^)	28.23 ± 5.26	25.7 ± 3.90	0.41
**HR-pQCT–Ultradistal Radius**			
Number of participants (n)	7	7	
Trabecular BMD (mgHA/mm^3^)	190.96 ± 37.35	179.47 ± 19.20	0.48
Cortical BMD (mgHA/mm^3^)	858.6 ± 73.44	865.37 ± 39.91	0.83
Cortical thickness (mm)	0.88 ± 0.21	0.86 ± 0.15	0.83
Trabecular number (1/mm)	2.18 ± 0.20	1.92 ± 0.21	0.36
**HR-pQCT–Ultradistal Tibia**			
Number of participants (n)	6	7	
Trabecular BMD (mgHA/mm^3^)	186.48 ± 52.15	178.82 ± 33.30	0.69
Cortical BMD (mgHA/mm^3^)	836.13 ± 75.55	897.0 ± 29.40	0.08
Cortical thickness (mm)	1.35 ± 0.29	1.33 ± 0.23	0.35
Trabecular number (1/mm)	1.97 ± 0.33	1.76 ± 0.18	0.35

## Discussion

The majority of bone research in subjects with type 2 diabetes mellitus (T2DM) has been conducted in postmenopausal women. A recent publication reported pronounced cortical disease in elderly, male type 2 diabetics, but, overall, there are only limited data about bone microarchitecture in men with T2DM. It thus remains to be determined whether and how bone morphology differs between diabetic men and women. Specifically addressing the issue of potential interactions between sex and T2DM, we recruited men and women with and without T2DM and performed HR-pQCT imaging of the ultradistal extremities.

High BMD without associated fracture risk reduction is recognized as a clinical key feature of diabetic bone disease [35, 36]. In the present study, we found a high trabecular number in men and women with T2DM, thereby supporting the results of other HR-pQCT studies in diabetic subjects [7, 15]. Trabecular hypertrophy has also been reported in pre-diabetic subjects with insulin resistance [37]. Trabecular rarefactions and increases in trabecular heterogeneity appear to be a feature of the later stages of diabetes-related bone disease [8, 38].

With regard to cortical morphology, we found significantly thicker radial cortices in diabetic men and women. Cortical hypertrophy in subjects with T2DM is in keeping with recent QCT data in diabetic subjects without fragility fractures [12]. HR-pQCT studies in elderly, diabetic women without fractures have reported cortical thickening, but this did not reach statistical significance [8, 11]. Supporting this phenotypic concept, cortical hypertrophy has also been found in prediabetic women [37].

Cortical deficits, on the contrary, appear to depend on the clinical characteristics of participants (e.g., with/without prevalent fragility fractures [8], race [14], presence of microvascular disease [39]), and thus, vary in extent from study to study. Interestingly, we found cortical hypertrophy to be partially attenuated in men (Fig 1). Despite recent reports of an unfavorable cortical microarchitecture in elderly, diabetic men [15], this finding was unexpected in our relatively young cohort. The unfavorable association between cortical morphology and male sex is surprising because, in the general (i.e., non-diabetic) population, men are at lower risk for osteoporosis and osteoporotic fractures than same-aged women [20]. From a clinical perspective, it thus remains to be determined whether cortical deficits translate into biomechanical deficits and high fracture risk in diabetic men and women.

At the radius, sex and T2DM tended to interact in terms of cross-sectional bone size. Specifically, we found small bone size in diabetic men, but not in diabetic women. Small cross-sectional bone size has been previously described in subjects with T2DM [12] and insulin resistance [37]. It thus appears to be another morphologic feature of diabetic bone disease. The relevance of small cross-sectional bone size lies in reduced biomechanical stability and higher susceptibility to fractures [40].

Supporting the validity of our dataset, the presence of a strong statistical main effect of sex on the skeleton was in line with the literature. Several HR-pQCT studies have found larger geometry and higher BMD in healthy men than in healthy women, confirming previous studies using different imaging tools, including central quantitative computed tomography (QCT), peripheral QCT, and DXA [41, 42]. In terms of microarchitecture, the male skeleton is known to exhibit greater trabecular number, greater trabecular thickness, and lower intertrabecular separation than the female skeleton [22, 43].

Bearing in mind the large number of HR-pQCT parameters and their multi-collinearity, we approached our dataset by factor-analysis and attempted to reduce the amount of data. From factor-analysis, we obtained four groups of HR-pQCT parameters with strong statistical intra-group connections. From a technical and pathophysiologic perspective, statistical group compositions were plausible. The first factor represented a group of parameters derived from trabecular BMD. The second factor contained microstructural indices of the trabecular compartment that are mathematically dependent on trabecular number. The third factor covered parameters driven by cortical features, including cortical thickness, cortical area, cortical bone mineral density, and total bone mineral density. The fourth factor contained geometric indices. Confirming the validity of this approach, the composition of factor-groups and the subsequent choice of representative post-hoc parameters (based on factor-weight) was identical for independent measurement sites (i.e., the radius and tibia).

With our diabetic participants being relatively young and free of fragility fractures, care must be taken when comparing them to participants from other diabetic cohorts investigated by HR-pQCT. In terms of the ‘hypertrophic’ bone pattern, the results of our female diabetics ranged between those found in pre-diabetic, hyperinsulinemic women [37] and postmenopausal women with manifest T2DM without fractures [7, 11]. Compared to elderly, male diabetic subjects studied by Paccou et al., our diabetic men exhibited a comparable trabecular phenotype and less pronounced—but manifest—cortical bone deficits [15].

While we consider the relatively young age of our participants and the statistical reduction of HR-pQCT parameters to be strengths of our study, there were several limitations that need to be specifically addressed. The sample size was small. Data were collected with two separate HR-pQCT devices, but they were cross-calibrated by a dedicated multicenter study (previously published) [27]. Identical acquisition protocols and evaluation protocols were used. In addition, we were able to demonstrate that there were no differences in HR-pQCT parameters for healthy (i.e.,non-diabetic) men scanned at either European site (Table 4).

Nevertheless, due to the current convention for scan-site definition (i.e., the use of fixed off-sets from the radiocarpal and tibiotarsal joint), the scan sites were minimally different in men and women. As given by the use of a fixed off-set, scans were acquired more distally in men than in women, with, e.g., cortical thickness slightly underrated. Conversely, bone size was slightly overrated in men when compared to women imaged with identical protocols. We did not include subjects with fragility fractures; thus, it is still unclear whether fragility fractures are linked to similar micro-structural pathologies in men and women.

In conclusion, our results suggest that skeletal hypertrophy is present in men and women with T2DM, but appears attenuated at the cortical sites of the lower extremities in diabetic men. Future investigations are needed to provide explanations for this sex-specific pattern of diabetic bone disease.

## References

[1] Schwartz AV, Sellmeyer DE, Ensrud KE, Cauley JA, Tabor HK, Schreiner PJ, et al Older women with diabetes have an increased risk of fracture: a prospective study. The Journal of clinical endocrinology and metabolism. 2001;86(1):32–8. Epub 2001/03/07. 10.1210/jcem.86.1.7139 11231974

[2] Janghorbani M, Van Dam RM, Willett WC, Hu FB. Systematic review of type 1 and type 2 diabetes mellitus and risk of fracture. American journal of epidemiology. 2007;166(5):495–505. Epub 2007/06/19. 10.1093/aje/kwm106 17575306

[3] de L II, van der Klift M, de Laet CE, van Daele PL, Hofman A, Pols HA. Bone mineral density and fracture risk in type-2 diabetes mellitus: the Rotterdam Study. Osteoporosis international: a journal established as result of cooperation between the European Foundation for Osteoporosis and the National Osteoporosis Foundation of the USA. 2005;16(12):1713–20. Epub 2005/06/09. 10.1007/s00198-005-1909-1 15940395

[4] Farr JN, Khosla S. Determinants of bone strength and quality in diabetes mellitus in humans. Bone. 2016;82:28–34. 10.1016/j.bone.2015.07.027 26211989 PMC4679576

[5] Schwartz AV, Hillier TA, Sellmeyer DE, Resnick HE, Gregg E, Ensrud KE, et al Older women with diabetes have a higher risk of falls: a prospective study. Diabetes Care. 2002;25(10):1749–54. Epub 2002/09/28. 12351472 10.2337/diacare.25.10.1749

[6] Vestergaard P. Discrepancies in bone mineral density and fracture risk in patients with type 1 and type 2 diabetes—a meta-analysis. Osteoporosis international: a journal established as result of cooperation between the European Foundation for Osteoporosis and the National Osteoporosis Foundation of the USA. 2007;18(4):427–44. Epub 2006/10/28. 10.1007/s00198-006-0253-4 17068657

[7] Burghardt AJ, Issever AS, Schwartz AV, Davis KA, Masharani U, Majumdar S, et al High-resolution peripheral quantitative computed tomographic imaging of cortical and trabecular bone microarchitecture in patients with type 2 diabetes mellitus. J Clin Endocrinol Metab. 2010;95(11):5045–55. Epub 2010/08/20. 10.1210/jc.2010-0226 20719835 PMC2968722

[8] Patsch JM, Burghardt AJ, Yap SP, Baum T, Schwartz AV, Joseph GB, et al Increased cortical porosity in type-2 diabetic postmenopausal women with fragility fractures. Journal of bone and mineral research: the official journal of the American Society for Bone and Mineral Research. 2012. Epub 2012/09/20. 10.1002/jbmr.1763 PMC3534818 22991256

[9] Karim L, Bouxsein ML. Effect of type 2 diabetes-related non-enzymatic glycation on bone biomechanical properties. Bone. 2016;82:21–7. 10.1016/j.bone.2015.07.028 26211993 PMC4679472

[10] Pietschmann P, Schernthaner G, Woloszczuk W. Serum osteocalcin levels in diabetes mellitus: analysis of the type of diabetes and microvascular complications. Diabetologia. 1988;31(12):892–5. Epub 1988/12/01. 3266486 10.1007/BF00265373

[11] Shu A, Yin MT, Stein E, Cremers S, Dworakowski E, Ives R, et al Bone structure and turnover in type 2 diabetes mellitus. Osteoporos Int. 2011. Epub 2011/03/23. 10.1007/s00198-011-1595-0 PMC3690650 21424265

[12] Heilmeier U, Carpenter DR, Patsch JM, Harnish R, Joseph GB, Burghardt AJ, et al Volumetric femoral BMD, bone geometry, and serum sclerostin levels differ between type 2 diabetic postmenopausal women with and without fragility fractures. Osteoporos Int. 2015;26(4):1283–93. 10.1007/s00198-014-2988-7 25582311

[13] Gaudio A, Privitera F, Battaglia K, Torrisi V, Sidoti MH, Pulvirenti I, et al Sclerostin levels associated with inhibition of the Wnt/beta-catenin signaling and reduced bone turnover in type 2 diabetes mellitus. J Clin Endocrinol Metab. 2012;97(10):3744–50. 10.1210/jc.2012-1901 22855334

[14] Yu EW, Putman MS, Derrico N, Abrishamanian-Garcia G, Finkelstein JS, Bouxsein ML. Defects in cortical microarchitecture among African-American women with type 2 diabetes. Osteoporos Int. 2014. 10.1007/s00198-014-2927-7 PMC4400116 25398431

[15] Paccou J, Ward KA, Jameson KA, Dennison EM, Cooper C, Edwards MH. Bone Microarchitecture in Men and Women with Diabetes: The Importance of Cortical Porosity. Calcif Tissue Int. 2016;98(5):465–73. 10.1007/s00223-015-0100-8 26686695

[16] Cohen A, Dempster DW, Muller R, Guo XE, Nickolas TL, Liu XS, et al Assessment of trabecular and cortical architecture and mechanical competence of bone by high-resolution peripheral computed tomography: comparison with transiliac bone biopsy. Osteoporos Int. 2010;21(2):263–73. Epub 2009/05/21. 10.1007/s00198-009-0945-7 19455271 PMC2908272

[17] Sornay-Rendu E, Boutroy S, Munoz F, Delmas PD. Alterations of cortical and trabecular architecture are associated with fractures in postmenopausal women, partially independent of decreased BMD measured by DXA: the OFELY study. J Bone Miner Res. 2007;22(3):425–33. Epub 2006/12/22. 10.1359/jbmr.061206 17181395

[18] Vico L, Zouch M, Amirouche A, Frere D, Laroche N, Koller B, et al High-resolution pQCT analysis at the distal radius and tibia discriminates patients with recent wrist and femoral neck fractures. J Bone Miner Res. 2008;23(11):1741–50. Epub 2008/07/31. 10.1359/jbmr.080704 18665795

[19] Liu XS, Cohen A, Shane E, Yin PT, Stein EM, Rogers H, et al Bone density, geometry, microstructure, and stiffness: Relationships between peripheral and central skeletal sites assessed by DXA, HR-pQCT, and cQCT in premenopausal women. Journal of bone and mineral research: the official journal of the American Society for Bone and Mineral Research. 2010;25(10):2229–38. Epub 2010/05/26. 10.1002/jbmr.111 PMC3128822 20499344

[20] Seeman E. Pathogenesis of bone fragility in women and men. Lancet. 2002;359(9320):1841–50. 10.1016/S0140-6736(02)08706-8 12044392

[21] Cooper C, Melton LJ 3rd. Epidemiology of osteoporosis. Trends in endocrinology and metabolism: TEM. 1992;3(6):224–9. 18407104 10.1016/1043-2760(92)90032-v

[22] Khosla S, Riggs BL, Atkinson EJ, Oberg AL, McDaniel LJ, Holets M, et al Effects of sex and age on bone microstructure at the ultradistal radius: a population-based noninvasive in vivo assessment. Journal of bone and mineral research: the official journal of the American Society for Bone and Mineral Research. 2006;21(1):124–31. Epub 2005/12/16. 10.1359/JBMR.050916 PMC1352156 16355281

[23] Marshall LM, Lang TF, Lambert LC, Zmuda JM, Ensrud KE, Orwoll ES, et al Dimensions and volumetric BMD of the proximal femur and their relation to age among older U.S. men. J Bone Miner Res. 2006;21(8):1197–206. 10.1359/jbmr.060506 16869717

[24] Sigurdsson G, Aspelund T, Chang M, Jonsdottir B, Sigurdsson S, Eiriksdottir G, et al Increasing sex difference in bone strength in old age: The Age, Gene/Environment Susceptibility-Reykjavik study (AGES-REYKJAVIK). Bone. 2006;39(3):644–51. 10.1016/j.bone.2006.03.020 16790372

[25] Patsch JM, Kohler T, Berzlanovich A, Muschitz C, Bieglmayr C, Roschger P, et al Trabecular Bone Microstructure and Local Gene Expression in Iliac Crest Biopsies of Men With Idiopathic Osteoporosis. Journal of Bone and Mineral Research. 2011;26(7):1584–92. 10.1002/jbmr.344 21308775

[26] Chaitou A, Boutroy S, Vilayphiou N, Munoz F, Delmas PD, Chapurlat R, et al Association between bone turnover rate and bone microarchitecture in men: the STRAMBO study. J Bone Miner Res. 2010;25(11):2313–23. 10.1002/jbmr.124 20499368

[27] Burghardt AJ, Hermannsson B, Pialat J, Boutroy S, Burrows M, Liu D, et al Cross-Site Reproducibility of Cortical and Trabecular Bone Density and Micro-Architecture Measurements by Hr-Pqct. Osteoporosis Int. 2010;21:45–6.

[28] Boutroy S, Bouxsein ML, Munoz F, Delmas PD. In vivo assessment of trabecular bone microarchitecture by high-resolution peripheral quantitative computed tomography. The Journal of clinical endocrinology and metabolism. 2005;90(12):6508–15. Epub 2005/09/29. 10.1210/jc.2005-1258 16189253

[29] Pialat JB, Burghardt AJ, Sode M, Link TM, Majumdar S. Visual grading of motion induced image degradation in high resolution peripheral computed tomography: Impact of image quality on measures of bone density and micro-architecture. Bone. 2011. Epub 2011/10/25. 10.1016/j.bone.2011.10.003 22019605

[30] Laib A, Hauselmann HJ, Ruegsegger P. In vivo high resolution 3D-QCT of the human forearm. Technol Health Care. 1998;6(5–6):329–37. Epub 1999/04/01. 10100936

[31] Hildebrand T, Ruegsegger P. A new method for the model-independent assessment of thickness in three-dimensional images. J Microsc-Oxford. 1997;185:67–75.

[32] Laib A, Ruegsegger P. Calibration of trabecular bone structure measurements of in vivo three-dimensional peripheral quantitative computed tomography with 28-microm-resolution microcomputed tomography. Bone. 1999;24(1):35–9. Epub 1999/01/23. 9916782 10.1016/s8756-3282(98)00159-8

[33] Davis KA, Burghardt AJ, Link TM, Majumdar S. The effects of geometric and threshold definitions on cortical bone metrics assessed by in vivo high-resolution peripheral quantitative computed tomography. Calcified Tissue Int. 2007;81(5):364–71. Epub 2007/10/24. 10.1007/s00223-007-9076-3 17952361

[34] Patsch JM, Zulliger MA, Vilayphou N, Samelson EJ, Cejka D, Diarra D, et al Quantification of lower leg arterial calcifications by high-resolution peripheral quantitative computed tomography. Bone. 2014;58:42–7. 10.1016/j.bone.2013.08.006 23954758 PMC4042679

[35] Schwartz AV, Vittinghoff E, Bauer DC, Hillier TA, Strotmeyer ES, Ensrud KE, et al Association of BMD and FRAX score with risk of fracture in older adults with type 2 diabetes. JAMA. 2011;305(21):2184–92. Epub 2011/06/03. 10.1001/jama.2011.715 21632482 PMC3287389

[36] Rubin MR, Patsch JM. Assessment of bone turnover and bone quality in type 2 diabetic bone disease: current concepts and future directions. Bone research. 2016;4:16001 10.1038/boneres.2016.1 27019762 PMC4802604

[37] Shanbhogue VV, Finkelstein JS, Bouxsein ML, Yu EW. Association between insulin resistance and bone structure in non-diabetic postmenopausal women. J Clin Endocrinol Metab. 2016:jc20161726. 10.1210/jc.2016-1726 PMC4971339 27243136

[38] Pritchard JM, Giangregorio LM, Atkinson SA, Beattie KA, Inglis D, Ioannidis G, et al Association of larger holes in the trabecular bone at the distal radius in postmenopausal women with type 2 diabetes mellitus compared to controls. Arthritis Care Res (Hoboken). 2012;64(1):83–91. 22213724 10.1002/acr.20602 PMC5096917

[39] Shanbhogue VV, Hansen S, Frost M, Jorgensen NR, Hermann AP, Henriksen JE, et al Compromised cortical bone compartment in type 2 diabetes mellitus patients with microvascular disease. Eur J Endocrinol. 2015;174(2):115–24. 10.1530/EJE-15-0860 26537860

[40] Bouxsein ML, Seeman E. Quantifying the material and structural determinants of bone strength. Best Pract Res Clin Rheumatol. 2009;23(6):741–53. 10.1016/j.berh.2009.09.008 19945686

[41] Baker JF, Davis M, Alexander R, Zemel BS, Mostoufi-Moab S, Shults J, et al Associations between body composition and bone density and structure in men and women across the adult age spectrum. Bone. 2013;53(1):34–41. 10.1016/j.bone.2012.11.035 23238122 PMC3552077

[42] Riggs BL, Melton Iii LJ 3rd, Robb RA, Camp JJ, Atkinson EJ, Peterson JM, et al Population-based study of age and sex differences in bone volumetric density, size, geometry, and structure at different skeletal sites. J Bone Miner Res. 2004;19(12):1945–54. 10.1359/JBMR.040916 15537436

[43] Macdonald HM, Nishiyama KK, Kang J, Hanley DA, Boyd SK. Age-related patterns of trabecular and cortical bone loss differ between sexes and skeletal sites: a population-based HR-pQCT study. Journal of bone and mineral research: the official journal of the American Society for Bone and Mineral Research. 2011;26(1):50–62. Epub 2010/07/02. 10.1002/jbmr.171 20593413

